# Identification of missense *MAB21L1* variants in microphthalmia and aniridia

**DOI:** 10.1002/humu.24218

**Published:** 2021-05-24

**Authors:** Sarah E. Seese, Linda M. Reis, Brett Deml, Christopher Griffith, Adi Reich, Robyn V. Jamieson, Elena V. Semina

**Affiliations:** ^1^ Department of Pediatrics and Children's Research Institute, Medical College of Wisconsin Children's of Wisconsin Milwaukee WI USA; ^2^ Department of Cell Biology, Neurobiology, and Anatomy The Medical College of Wisconsin Milwaukee Wisconsin USA; ^3^ Department of Pediatrics University of South Florida Tampa Florida USA; ^4^ GeneDx Gaithersburg Maryland USA; ^5^ Eye Genetics Research Unit, Sydney Children's Hospitals Network and Children's Medical Research Institute University of Sydney Sydney New South Wales Australia; ^6^ Department of Ophthalmology and Visual Sciences Medical College of Wisconsin Milwaukee Wisconsin USA; ^7^ Present address: PreventionGenetics Marshfield Wisconsin USA

**Keywords:** aniridia, coloboma, MAB21L1, microphthalmia, rescue

## Abstract

Microphthalmia, coloboma, and aniridia are congenital ocular phenotypes with a strong genetic component but often unknown cause. We present a likely causative novel variant in *MAB21L1*, c.152G>T p.(Arg51Leu), in two family members with microphthalmia and aniridia, as well as novel or rare compound heterozygous variants of uncertain significance, c.184C>T p.(Arg62Cys)/c.‐68T>C, and c.658G>C p.(Gly220Arg)/c.*529A>G, in two additional probands with microphthalmia, coloboma and/or cataracts. All variants were predicted as damaging by in silico programs. In vitro studies of coding variants revealed normal subcellular localization but variable stability for the corresponding mutant proteins. In vivo complementation assays using the zebrafish *mab21l2* ^Q48Sfs*5^ loss‐of‐function line demonstrated that though overexpression of wild‐type *MAB21L1* messenger RNA (mRNA) compensated for the loss of *mab21l2*, none of the coding variant mRNAs produced a statistically significant rescue, with p.(Arg51Leu) showing the highest degree of functional deficiency. Dominant variants in a close homolog of *MAB21L1, MAB21L2*, have been associated with microphthalmia and/or coloboma and repeatedly involved the same Arg51 residue, further supporting its pathogenicity. The possible role of p.(Arg62Cys) and p.(Gly220Arg) in microphthalmia is similarly supported by the observed functional defects, with or without an additional impact from noncoding *MAB21L1* variants identified in each patient. This study suggests a broader spectrum of *MAB21L1*‐associated disease.

## Graphical abstract

MAB21L1 alleles in microphthalmia and aniridia.

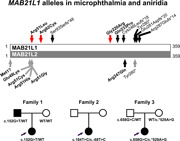

## 1 INTRODUCTION

Developmental ocular disorders have complex genetic etiologies due to the intricate and tightly controlled genetic networks involved in eye development (Skalicky et al., 2013). Microphthalmia, anophthalmia, and coloboma (MAC) are rare congenital malformations of the eye involving a small eye, absence of an eye, and gap in ocular structures, respectively (Gregory‐Evans et al., 2004; Verma & Fitzpatrick, 2007). Over 80 genes have been published in association with MAC phenotypes (Reis & Semina, 2015). However, about 50% of patients lack a confirmed genetic diagnosis, suggesting novel genes have yet to be discovered (Plaisancie et al., 2016).

Aniridia is a panocular disorder with its primary feature being a partial or complete absence of the iris, but also including lens opacities, glaucoma, keratopathy, foveal and optic nerve hypoplasia, strabismus, ptosis, and fibrosis syndrome (Hall et al., 2019; Hingorani et al., 2012; Lim et al., 2017). Up to 90% of cases with aniridia can be explained by loss‐of‐function mutations in the *PAX6* gene (Hingorani et al., 2012); rarely, disruption of *FOXC1, PITX2*, and other genes have been identified as causative (Hall et al., 2019; Hingorani et al., 2012). However, there is a small portion of the aniridia population that remains genetically unexplained.

The Male‐Abnormal 21‐Like gene *MAB21L2* is a recently identified factor involved in human MAC‐spectrum disorders, where both dominant and recessive missense alleles have been recognized as causative in eight unrelated families (Aubert‐Mucca et al., 2020; Deml et al., 2015; Horn et al., 2015; Patel et al., 2018; Rainger et al., 2014). In four out of seven dominant families, the pathogenic variant is a missense allele affecting residue 51 of the resulting protein. Similarly, a mouse *Mab21l2* model with a heterozygous p.(Arg51Cys) mutant allele (identical to two of the affected human patients (Horn et al., 2015; Rainger et al., 2014), further demonstrated the importance of this residue, resulting in defects in early ocular development including rudimentary and mispositioned optic cup, undetectable optic stalk, abnormalities of the retinal pigment epithelium, and failure to induce a lens placode (Tsang et al., 2018). Additional null/loss‐of‐function animal models have displayed MAC‐spectrum defects in mice (Yamada et al., 2004) and zebrafish (Deml et al., 2015; Gath & Gross, 2019; Hartsock et al., 2014; Wycliffe et al., 2020).

*MAB21L1*, a closely related family member to *MAB21L2*, has also been implicated in human disease. Homozygous *MAB21L1* variants have been reported in six unrelated families exhibiting cerebello‐oculo‐facio‐genital syndrome, with five of these variants resulting in premature truncation of the protein (Bruel et al., 2017; Rad et al., 2019). Ocular abnormalities included corneal dystrophy/opacities, nystagmus, strabismus, dry eye, pigment granularity, retinal degeneration, optic atrophy, buphthalmos, and cataracts (Bruel et al., 2017; Rad et al., 2019). A null mouse model for *Mab21l1* likewise exhibits embryonic ocular defects, though more severe than the published human phenotype. Abnormalities include microphthalmia, malformed retina, and retinal pigment epithelium, along with aphakia, thickened cornea, and absent iris (Yamada et al., 2003).

The precise protein function(s) of the MAB21L family is unknown. A possible role in transcriptional regulation has been suggested (Baldessari et al., 2004) with nuclear localization (Mariani et al., 1999) and a mild affinity for nucleic acid shown in vitro (de Oliveira Mann et al., 2016; Rainger et al., 2014).

Here, we report three families with unique *MAB21L1* variants exhibiting ocular phenotypes including MAC‐spectrum and aniridia. Functional studies of the proteins associated with coding variants revealed differences from wild‐type MAB21L1. Thus, this study suggests a phenotypic expansion for *MAB21L1*‐associated human disease.

## 2 MATERIALS AND METHODS

### 2.1 Editorial policies and ethical considerations

Human studies conformed to the US Federal Policy for the Protection of Human Subjects and were approved by the Children's Hospital of Wisconsin Institutional Review Board and the Sydney Children's Hospitals Network Research Ethics Committee, with written informed consent obtained from all participating individuals and/or their legal representatives.

### 2.2 Human DNA screening and in silico variant analyses

The *MAB21L1* variant in Individual 1 was initially identified through clinical exome sequencing using the previously published protocol (Guillen Sacoto et al., 2020) and matched to the study through the Matchmaker Exchange GeneMatcher node (Philippakis et al., 2015; Sobreira et al., 2015) followed by enrollment and research exome sequencing and analysis; the *MAB21L1* variants in Individuals 2 and 3 were identified through research exome sequencing and analysis using previously described methods (Deml et al., 2016; Ma et al., 2020). Variants in known ocular genes were ruled out in all three families. All variants were confirmed via Sanger sequencing of the *MAB21L1* coding region by amplification of a 1405‐base pair (bp) product using the flanking primers F‐5ʹ‐CCGAAAGGCATTTTTGATCC‐3ʹ, R‐5ʹ‐TCCGCTTCCCCTACTTTTTC‐3ʹ, and also internal primers F‐5ʹ‐AGATCACGCCGGCCTTTA‐3ʹ, R‐5ʹ‐ACCCAGGCGTCGCTCTC‐3ʹ. Polymerase chain reaction (PCR) amplicons were sequenced as previously described (Deml et al., 2015) or through Functional Biosciences™ DNA Sequencing Services. Parental samples were analyzed using the same protocol. To determine relative positions of variants identified in Individual 2, the amplified 1405‐bp product was cloned into a pCR®II‐TOPO® plasmid, which was followed by sequencing of 32 independent clones; 18 clones contained c.184C>T p.(Arg62Cys) allele and wild‐type 5ʹ‐UTR (untranslated region) sequence and 14 clones contained c.‐68T>C 5ʹ‐UTR variant and wild‐type coding region sequence indicating their *trans* configuration.

The following information was collected for the identified variants: Frequency in the general, ethnically matched population using gnomAD (Karczewski et al., 2020), predicted effect on protein function, and amino acid conservation (via dbNSFP (X. Liu et al., 2013) accessed through Ensembl Variant Effect Predictor (McLaren et al., 2016)) and nucleotide conservation (using UCSC Genome Browser; (Kent et al., 2002)) with results obtained for the following tools—SIFT (Sim et al., 2012), Polyphen2 (Adzhubei et al., 2010), MutationTaster (Schwarz et al., 2014), MutationAssessor (Reva et al., 2011), FATHMM‐MKL (Shihab et al., 2014), CADD PHRED (Rentzsch et al., 2019), REVEL (Ioannidis et al., 2016), GERP++RS (Davydov et al., 2010), and PhyloP100wayAll (Pollard et al., 2010). The reported score ranges and pathogenic criteria for the above programs are as follows: For CADD PHRED, scores over 20 and 30 have been found to be the top 1% and 0.1% most damaging variants in the genome, respectively (Kircher et al., 2014); for REVEL, the range is 0 to 1 with 75.4% of disease‐causing variants having a score >0.5 (Ioannidis et al., 2016); for nucleotide conservation, GERP++RS scores range from −12.3 to 6.17 (max, and most conserved) and PhyloP100way vertebrate scores range −20.0 to 10.003 (max, and most conserved). To highlight conservation, nucleotide alignments were generated using UCSC Multiz Alignments of 100 Vertebrates; protein alignments were generated with Kalign multiple sequence alignment tool using the following sequences: Human MAB21L1 (NP_005575.1), human MAB21L2 (NP_006430.1), mouse Mab21l1 (NP_034880.1), chicken Mab21l1 (NP_989864.1), zebrafish mab21l1 (NP_694506.2), and *Caenorhabditis elegans* mab‐21 (NP_497940.2).

Additional in silico analyses were performed for the *MAB21L1* UTR variants (c.‐68T>C and c.*529A>G) to assess possible functional impacts. To determine changes to minimum free energy and RNA secondary structure, both 5ʹ‐ and 3ʹ‐ UTR DNA sequences carrying wild‐type or variant alleles were submitted to the RNAfold 2.4.17 Webserver, accessed through the Vienna RNA Websuite 2.0 (Lorenz et al., 2011). To assess changes to microRNA target prediction, wild‐type, and the variant 3ʹ‐UTR sequence was submitted to MicroSNiPer (Barenboim et al., 2010) with a minimum seed length constraint of seven base pairs. In addition, the PolymiRTS Database 3.0, a database of human SNPs affecting predicted microRNA (miRNA) target sites (Bhattacharya et al., 2014), was searched for the 3ʹ‐UTR variant, rs1775984, to determine overlapping predicted miRNA sites. PolymiRTS calculated the strength of the predicted miRNA site and provided a conservation score, which was determined based on the number of vertebrate genomes in which the miRNA site is present, and context+ score change (a ranking of miRNA target predictions) (Garcia et al., 2011). To determine variant effects on RNA binding protein (RBP) binding sites, wild‐type and the variant 5ʹ‐ and 3ʹ‐UTR sequences were submitted to RBPmap version 1.1 (Paz et al., 2014) to identify RBP motifs. Predicted motifs containing the affected nucleotide were assessed for lost/gained interactions between wild‐type or mutant sequence and RBPs.

In silico protein modeling was executed for the MAB21L1 wild‐type and MAB21L1‐p.(Arg51Leu), MAB21L1‐p.(Arg62Cys), and MAB21L1‐p.(Gly220Arg) proteins. The wild‐type MAB21L1 crystal structure has been previously solved and, thus, the full‐length MAB21L1 structure pdb file (PDB ID: 5EOG) (de Oliveira Mann et al., 2016) was utilized (RCSB Protein Data Bank). To predict changes to the structure invoked by the three missense mutations, the altered protein sequence was submitted to I‐TASSER (Yang & Zhang, 2015) and the corresponding pdb file downloaded (predicted model #1). Files were uploaded into PyMOL (The PyMOL Molecular Graphics System, Version 2.0; Schrödinger, LLC) where labeled images of protein structures were created. α‐Helices and β‐sheets were named as previously described (de Oliveira Mann et al., 2016).

### 2.3 Western blot analysis, immunofluorescence, and enzyme‐linked immunosorbent assay (ELISA) experiments

For protein expression experiments, MAB21L1 wild‐type and mutant constructs for the p.(Arg51Leu), p.(Arg62Cys), and p.(Gly220Arg) were developed. To do so, an N‐terminal FLAG‐tagged human *MAB21L1* (NM_005584.4) clone in a pEZ‐M11 vector was obtained (GeneCopoeia™). To generate mutants, site‐directed mutagenesis was performed using the QuikChange Lightning Site‐Directed Mutagenesis Kit (Agilent Technologies). High‐performance liquid chromatography (HPLC) purified primers were as follows: For c.152G>T p.(Arg51Leu), s‐5ʹ‐GAGAGCTGATGAACAGCGGCTCCTGCACT‐3ʹ, as‐5ʹ‐AGTGCAGGAGCCGCTGTTCATCAGCTCTC‐3ʹ; for c.184C>T p.(Arg62Cys), s‐5ʹ‐GCCCTCGTAGCAATTGTCCATCTCGTTGAGAGA‐3ʹ, as‐5ʹ‐TCTCTCAACGAGATGGACAATTGCTACGAGGGC‐3ʹ; and for c.658G>C p.(Gly220Arg), s‐5ʹ‐GAGCTCTGCTTGCGGGCCAAGGAGTGG‐3ʹ, as‐5ʹ‐CCACTCCTTGGCCCGCAAGCAGAGCTC‐3ʹ. Transformed colonies were selected and plasmids isolated using an Invitrogen™ PureLink™ Quick Plasmid Miniprep Kit (Thermo Fisher Scientific) and sequenced with the following primers: F‐5ʹ‐CAGCCTCCGGACTCTAGC‐3ʹ, R‐5ʹ‐TAATACGACTCACTATAGGG‐3ʹ.

Then, 2.5 μg of N‐terminal FLAG‐tagged MAB21L1 wild‐type and mutant constructs, were transfected into Human Lens Epithelial (HLE‐B3) cells (ATCC®) using Invitrogen™ Lipofectamine™ 2000 Transfection Reagent (Thermo Fisher Scientific) in Opti‐MEM (Thermo Fisher Scientific). Cells were cultured in Gibco™ minimal essential medium (Thermo Fisher Scientific) with 20% fetal bovine serum (Millipore Sigma‐Aldrich), 1× l‐glutamine (Thermo Fisher Scientific), and 1× sodium pyruvate (Thermo Fisher Scientific). Forty‐eight hours posttransfection, cells were collected in 1× phosphate‐buffered saline (PBS). To obtain whole‐cell lysates for Western blot and ELISA analysis, pelleted cells were resuspended in 1% Triton™ X‐100 (Millipore Sigma‐Aldrich) with 100× protease inhibitor (Millipore Sigma‐Aldrich).

For Western blot analysis, samples were denatured by adding 4× Laemmli sample buffer (Bio‐Rad Laboratories, Inc.), boiled at 95°C for 5  min, and run on a 10% Criterion™ Tris‐HCl Precast Gel (Bio‐Rad Laboratories, Inc.). Membranes were incubated with mouse 1:1000 anti‐FLAG (Millipore Sigma‐Aldrich) or 1:1000 rabbit anti‐βactin for normalization (GeneTex) overnight, and the following day with the corresponding secondary antibody, either 1:2000 goat anti‐mouse horseradish peroxidase (HRP) conjugate (Thermo Fisher Scientific) or 1:2000 goat anti‐rabbit HRP conjugate (Thermo Fisher Scientific). For detection, SuperSignal™ West Pico or Femto Maximum Sensitivity chemiluminescent substrates were used (Thermo Fisher Scientific).

For ELISA semiquantitative protein expression analysis, a DYKDDDDK‐Tag Detection ELISA Kit (Cayman Chemical) was used. Biological samples (from three separate transfections) were run in duplicate. Data were analyzed using elisaanalysis.com with four‐parameter regression analysis and plotted in GraphPad Prism 9 (GraphPad). Statistical significance was determined using an unpaired samples *t‐test* and a *p* < .05.

Immunofluorescence experiments were used to determine protein localization. The transfection protocol was as described above with 7.5 μg wild‐type and mutant N‐terminally tagged MAB21L1 constructs into HLE‐B3 cells. Cells were fixed with 1:1 methanol/acetone permeabilized with 1% Triton X‐100 and blocked with 10% donkey serum in 1× PBS, followed by overnight incubation with 1:100 mouse anti‐FLAG primary antibody at 4°C. The next day, cells were incubated with 1:1000 donkey anti‐mouse Alexa Fluor 488 secondary antibody (Thermo Fisher Scientific) and stained with Invitrogen™ 4ʹ,6‐diamidino‐2‐phenylindole, dihydrochloride (DAPI) (Thermo Fisher Scientific).

### 2.4 Animal care and use

The care and use of zebrafish have been approved by the Institutional Animal Care and Use Committee at the Medical College of Wisconsin in compliance with the US National Research Council's Guide for the Care and Use of Laboratory Animals, the US Public Health Service's Policy on Humane Care and Use of Laboratory Animals, and Guide for the Care and Use of Laboratory Animals. All breeding and housing were conducted as previously described (Y. Liu & Semina, 2012). Developmental stages were determined as previously described by hours post fertilization (hpf) and morphology (Kimmel et al., 1995).

### 2.5 RNA complementation assay

For mRNA complementation assays, *MAB21L1* wild‐type and mutant (p.(Arg51Leu), p.(Arg62Cys), and p.(Gly220Arg)) constructs were developed. To do so, a human *MAB21L1* (NM_005584.4) clone in a pCR®II‐TOPO® vector was used to generate mutants by performing site‐directed mutagenesis using the QuikChange Lightning Site‐Directed Mutagenesis Kit (Agilent Technologies). HPLC purified primers for the *MAB21L1* mutant constructs are as described above. Transformed colonies were selected, plasmids isolated using Invitrogen™ PureLink™ Quick Plasmid Miniprep Kit (Thermo Fisher Scientific) and sequenced with the following primers: F‐5ʹ‐GTAAAACGACGGCCAG‐3ʹ, R‐5ʹ‐CAGGAAACAGCTATGAC‐3ʹ. Plasmids were then linearized with the restriction enzyme HindIII (Millipore Sigma‐Aldrich) and a DNA Clean and Concentrator kit was used as needed (ZymoResearch). mRNA was synthesized from the linearized plasmid using the Invitrogen™ mMESSAGE mMACHINE™ T7 Transcription Kit (Thermo Fisher Scientific) and the Invitrogen™ Poly(A) Tailing Kit (Thermo Fisher Scientific). mRNA was purified using an RNA Clean & Concentrator Kit (ZymoResearch) or phenol‐chloroform purification. Concentration and purity were measured using a NanoDrop™ 1000 Spectrophotometer (Thermo Fisher Scientific) and integrity was assessed with agarose gel electrophoresis. Four hundred picograms of wild‐type or mutant mRNA were injected using a Drummond Nanoject II instrument (Drummond Scientific), into embryos from a *mab21l2* c.141_153del p.(Gln48Serfs*5) heterozygous cross (Deml et al., 2015). At 24 hpf, when the mutant phenotype is clearly observable in homozygous *mab21l2* ^Q48Sfs*5^ embryos, injected offspring were examined for the presence or absence of the previously described ocular phenotype, and the proportion of normal embryos was determined. Graphs were created using GraphPad Prism 9. Statistical significance was determined using an unpaired samples *t‐test* and a *p* < .05.

## 3 RESULTS

### 3.1 Identification of *MAB21L1* variants in three families with congenital ocular disease

Examination of exome sequencing data in genetically unexplained families with MAC phenotypes identified several new variants of interest within the *MAB21L1* gene, including a likely causative heterozygous missense variant in one family with dominant transmission (Individuals 1A and 1B) and four additional variants of uncertain significance in two unrelated families, with one coding (missense) and one noncoding allele in *trans* in each affected proband.

**Figure 1:**
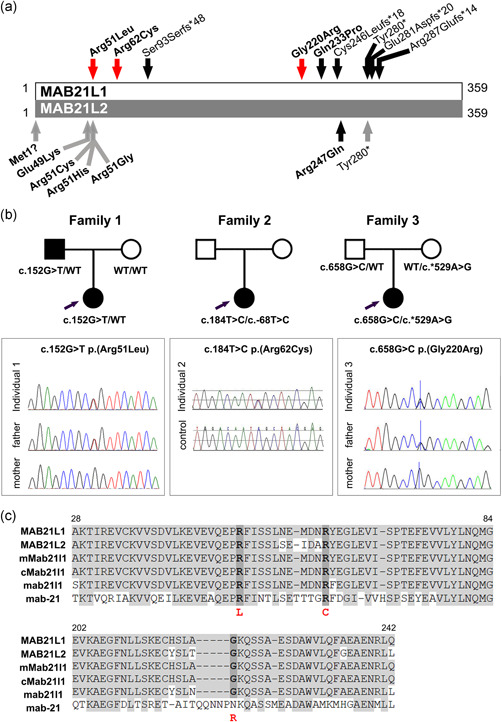
*MAB21L1*variant details. (a) Schematic of MAB21L1 (top) and MAB21L2 (bottom) proteins. Newly identified heterozygous MAB21L1 coding variants are indicated by a red arrow; previously reported MAB21L1 and MAB21L2 recessive variants are indicated with a black arrow; previously reported MAB21L2 dominant variants are indicated with a gray arrow. Missense variants are bolded. (b) Pedigrees for Families 1–3 indicating *MAB21L1* genotype and Sanger sequencing traces for the identified coding variants. (c) Alignment of MAB21L1 and related proteins showing conservation at and around the Arg51Leu, Arg62Cys, and Gly220Arg variants. Identical amino acids are shaded in gray; positions of variant amino acids are indicated with dark gray. Human MAB21L1 (NP_005575.1), human MAB21L2 (NP_006430.1), mouse Mab21l1 (NP_034880.1), chicken Mab21l1 (NP_989864.1), zebrafish mab21l1 (NP_694506.2), and *Caenorhabditis elegans* mab‐21 (NP_497940.2) are shown

Individual 1A is a 3‐year‐old female child from the Dominican Republic (Black/Hispanic ancestry) with bilateral microphthalmia, aniridia, microcornea, microspherophakia, and nystagmus; she is otherwise non‐dysmorphic and developmentally normal. Clinical sequencing and deletion/duplication analysis of *PAX6, FOXC1, PITX2*, and *CYP1B1*, as well as a clinical microphthalmia/anophthalmia gene panel, did not reveal causative variants. Whole‐exome sequencing identified a heterozygous missense variant in *MAB21L1*, c.152G>T p.(Arg51Leu) (Figure 1 and Table 1), and repeat research exome analysis confirmed this and did not identify any other pathogenic variants. The variant was predicted to be damaging by 5/5 programs and combined scores from REVEL (0.682) and CADD PHRED (29.6) also supported pathogenicity (Table 1). The arginine residue at position 51 is highly conserved with a GERP++RS score of 5.66 and a PhyloP score of 7.78; the identified variant is novel (not present in ~250,000 alleles in the general population). Family history revealed that her father (Individual 1B) also exhibits bilateral microphthalmia, aniridia, ectopia lentis, and microcornea; research exome analysis and Sanger sequencing identified the same heterozygous c.152G>T p.(Arg51Leu) variant in the father and wild‐type *MAB21L1* alleles in the unaffected mother (Figure 1b). Interestingly, variants affecting the same conserved Arg51 residue in a close homolog of MAB21L1, MAB21L2, are known to cause dominant MAC (Deml et al., 2015; Horn et al., 2015; Rainger et al., 2014), further supporting the causality of the identified *MAB21L1* c.152G>T p.(Arg51Leu) allele.

**Table 1:** Summary of new and previously reported *MAB21L1* variants

ID	Age; ancestry	DNA change^a^	Protein change	gnomAD^b^	Predicted effect^c^												Phenotype	
					SIFT	PP	MT	MA	FM	REV	CADD	GERP	Phy	RNA fold	Micro	RBP	Eye	Other
Heterozygous and compound heterozygous variants																		
Individual 1A (this report)	3 years; Black/Hispanic	c.152G>T	p.Arg51Leu	0/~250,000	D	D	D	D	D	0.68	29.6	5.66	7.78	–	–	–	B microphthalmia, microcornea, aniridia, microspherophakia, nystagmus	NR
Individual 1B (this report)	25 years; Black/Hispanic	c.152G>T	p.Arg51Leu	0/~250,000	D	D	D	D	D	0.68	29.6	5.66	7.78	–	–	–	B microphthalmia, microcornea, aniridia, ectopia lentis	NR
Individual 2 (this report)	6 months; South Asian	c.184C>T	p.Arg62Cys	2/30,616	T	D	D	D	D	0.59	31	5.66	9.87	–	–	–	L microphthalmia, coloboma; R cataract	NR
		c.‐68T>C	–	0/~31,000	–	–	–	–	D	–	–	3.36	1.01	U	–	A		
Individual 3 (this report)	2 years; White	c.658G>C	p.Gly220Arg	1/112,548	T	D	D	D	D	0.45	26.2	5.76	7.80	–	–	–	B microphthalmia, coloboma	NR
		c.*529A>G	–	715/15,432	–	–	–	–	D	–	–	5.24	2.20	U	A	A		
Homozygous variants																		
Bruel et al. (2017)	7 years; Algerian	c.735dup	p.Cys246Leufs	0/~250,000	Premature truncation									–	–	–	Corneal dystrophy, buphthalmos, strabismus, nystagmus	FD, SA, GD, CM
Rad et al. (2019): Fam 1	5 years, 7 years, 17 years 7 months; Persian	c.841del	p.Glu281Aspfs	0/~250,000	Premature truncation									–	–	–	Corneal dystrophy, strabismus, nystagmus	FD, SA, AL, GD, CM
Rad et al. (2019): Fam 2	26 months; Persian	c.698A>C	p.Gln233Pro	0/~280,000	D	T	D	T	D	0.33	25.2	5.76	8.0	–	–	–	Corneal dystrophy, nystagmus, retinal dystrophy, optic atrophy	FD, SA, CM
Rad et al. (2019): Fam 3	2 years; Lebanese Shia	c.279_286 del	p.Ser93Serfs	0/~250,000	Premature truncation									–	–	–	Corneal dystrophy	FD, AL, GD, CM
Rad et al. (2019): Fam 4	18 years 9 m; Turkish	c.859del	p.Arg287Glufs	0/~250,000	Premature truncation									–	–	–	Corneal dystrophy, nystagmus	FD, SA, GD, CM
Rad et al. (2019): Fam 5	10 years, 16 years, 6 months, 12 months; Turkish	c.840C>G	p.Tyr280*	0/~250,000	Premature truncation									‐	‐	‐	Corneal dystrophy, nystagmus	FD, SA, AL, GD, CM

Abbreviations: A, altered; AL, absent labia majora; B, bilateral; CM, cerebellar malformation; D, damaging; FD, facial dysmorphism; GD, global development delay; L, left; NR, none reported; R, right; SA, scrotal agenesis; T, tolerated; U, unaffected; ‐, not applicable or not assessed.

^a^ RefSeq NM_005584.4.

^b^ gnomAD frequency for the relevant population is indicated.

^c^ programs utilized: PP, PolyPhen; MT, Mutation Taster; MA, Mutation assessor; FM, FATHMM‐MKL; REV, REVEL; CADD, CADD PHRED; GERP, GERP++ RS; Phy, PhyloP; Micro, MicroSNiPer and PolymiRTS; RBP, RBPmap.

Individual 2, a 6‐month‐old South Asian (Indian) female diagnosed with microphthalmia and optic disc coloboma in the left eye and isolated congenital cataract in the right eye, was found to have a heterozygous missense variant in *MAB21L1*, c.184C>T p.(Arg62Cys) (Figure 1 and Table 1). No systemic abnormalities were noted. The amino acid substitution was predicted to be likely damaging by 4/5 prediction programs and had REVEL (0.585) and CADD PHRED (31) scores suggesting pathogenicity (Table 1). Again, the arginine residue at position 62 was found to be highly conserved with a GERP++RS score of 5.66 and a PhyloP score of 9.87 (Table 1). However, the variant was found to be present in the general population, albeit at a very low frequency (2/30,616 alleles, 0.0065% in the ethnically matched South Asian population). Additionally, the patient was found to have a novel (0/~31,000 alleles) heterozygous noncoding variant in the 5ʹ‐UTR of the *MAB21L1* gene, c.‐68T>C. The patient's parents were unaffected but unavailable for further testing; however, the variant alleles were determined to be positioned in *trans* through PCR amplification, cloning, and sequencing of multiple copies of the region encompassing both alleles.

Individual 3 is a 2‐year‐old white (Australian) female patient diagnosed with bilateral colobomatous microphthalmia and also identified to have a heterozygous missense variant in *MAB21L1*, c.658G>C p.(Gly220Arg) (Figure 1 and Table 1). The amino acid substitution was predicted to be likely damaging by 4/5 programs and had a CADD PHRED score of 26.2, suggesting pathogenicity (Table 1); the REVEL score was 0.45 (~25% of pathogenic variants have a score below 0.5 (Ioannidis et al., 2016)). The glycine residue at position 220 was found to be highly conserved with a GERP++RS score of 5.76 and a PhyloP score of 7.80. The variant was found to be present in 1/112,548 alleles in the ethnically matched European (non‐Finnish) population (0.00178%) in gnomAD. Additionally, the patient was found to have a heterozygous noncoding variant in the 3ʹ‐UTR of the *MAB21L1* gene, c.*529A>G, which is present in 715 of 15,432 alleles in the European (non‐Finnish) population (4.6%; 18 homozygotes reported). Both parents are unaffected; examination of parental samples identified the coding variant in the father's sample and the noncoding allele in the mother, indicating *trans* configuration for these alleles in the patient.

### 3.2 In silico analysis of coding and noncoding alleles

For in silico modeling of the wild‐type and mutant MAB21L1 proteins, iTASSER and Pymol software were utilized (Figure S1). The wild‐type protein structure is described as two‐lobed, containing an N‐terminal and C‐terminal lobe, with an α‐helix spine (α1) spanning the two; the N‐terminal lobe is surface‐accessible and contains a subdomain with structural homology to the catalytic nucleotidyltransferase (NTase) core domain of cyclic GMP–AMP synthase (cGAS) (de Oliveira Mann et al., 2016). The arginine 51 residue is located just outside of the α1 spine; the arginine 62 residue is located in the same linker region between α1 and β1 and the glycine residue at position 220 is also found in a linker region between β8 and β9.

Comparing the p.(Arg51Leu) predicted structure to the established wild‐type, there appears to be a loss of α3 in the N‐terminal lobe. For p.(Arg62Cys), the most notable difference was the loss of β8 and β9 within the NTase core subdomain of the N‐terminal lobe. There is also a subtle distortion of the α1 spine. Interestingly, previous work in co‐crystallizing MAB21L1 with a CTP moiety found that CTP interacted directly with the Arg62 residue in a positively charged pocket, denoted the “ligand‐binding pocket” (de Oliveira Mann et al., 2016). Although no nucleotidyltransferase catalytic activity has been determined for this protein or its family members (de Oliveira Mann et al., 2016; Rainger et al., 2014), changes to this residue and the positively charged side chain may affect its ability to form ligand interactions. Finally, comparing the p.(Gly220Arg) predicted structure to wild‐type, there was a gain of a small α‐helix between β8 and β9 of the NTase core domain near the N‐terminal lobe as well as a downward shift in the position of α8. These identified structural deviations have the possibility to translate to functional effects such as altered interactions.

To investigate noncoding variants, in silico analyses were conducted to assess the effects on the upstream open reading frame (uORF), RNA secondary structure and minimum free energy, miRNA target sites, and RBP motifs. No effect on the uORF (for the c.‐68T>C variant) and no secondary structure changes (for either the c.‐68T>C or c.*529A>G) were predicted, with little or no effect on minimum free energy (−156.90 kcal/mol for wild‐type 5ʹ‐UTR, −156.90 kcal/mol for c.‐68T>C; −239.64 kcal/mol for wild‐type 3ʹ‐UTR, −239.94 kcal/mol for c.*529A>G) in comparison to corresponding wild‐type sequences. As miRNAs typically bind to sites within the 3ʹ‐UTR, potential target sites were assessed in wild‐type and the variant 3ʹ‐UTR sequence (Grimson et al., 2007). This revealed several target sites that were either disrupted or created by the c.*529A>G variant (Table S1). In addition, several RBP motifs in both the 5ʹ‐ and 3ʹ‐UTR were predicted to be affected: c.‐68T>C disrupted a potential SRSF3 motif and created an RBM6 motif; c.*529A>G disrupted predicted motifs for HNRNPL, IGF2BP2, RBM41, and SRSF3 RNA binding factors and generated a new sequence expected to bind SRSF5 (Table S2). These RNA‐binding proteins (or their closely related family members) have demonstrated important roles in RNA‐regulation (including splicing, export, stability, polyadenylation, and translation) (Cao et al., 2018; Jain et al., 2012; Oberdoerffer et al., 2008; Rothrock et al., 2005; Sutherland et al., 2005; Twyffels et al., 2011; Zhong et al., 2009; Zhou et al., 2020); in addition, SRSF3 and HNRNPL showed associations with ocular disease, mainly glaucoma (Jain et al., 2012; Schmitt et al., 2020). Finally, in silico evaluations using FATHMM‐MKL predicted deleterious effects, and GERP++RS and PhyloP indicated nucleotide conservation for both 5ʹ‐ and 3ʹ‐UTRs (Table 1 and Figure S2). Therefore, though the mechanisms remain unclear, it is possible the noncoding variants in Individuals 2 and 3 could be contributing to disease through disruption of miRNA and RBP target sites, and thus, regulatory activities upon the *MAB21L1* mRNA.

### 3.3 Expression and localization of MAB21L1 wild‐type and mutant proteins

**Figure 2:**
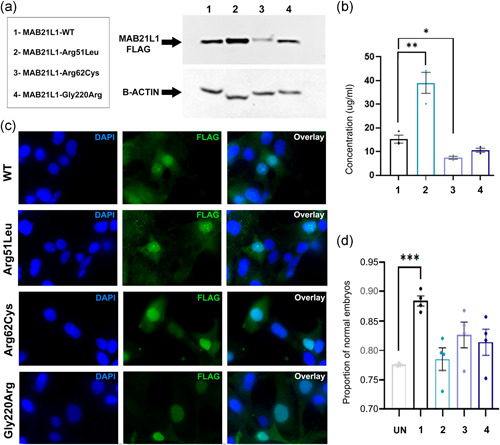
Functional analyses of MAB21L1 and variant proteins. (a) Western blot analysis. Western blot analysis of N‐terminal FLAG‐tagged MAB21L1 wild‐type and Arg51Leu, Arg62Cys, and Gly220Arg variants. Constructs were expressed in HLE‐B3 cells. β‐actin was used as a loading control. The proteins correspond to their expected molecular weight (~41kDa MAB21L1 and ~42kDa β‐actin). (b) Enzyme‐linked immunosorbent assay. N‐terminal FLAG‐tagged MAB21L1 wild‐type and Arg51Leu, Arg62Cys, and Gly220Arg variants were transfected into HLE‐B3 cells. Cell lysates were assessed for FLAG‐tagged protein expression; protein levels of Arg51Leu and Arg62Cys were found to be significantly affected. (c) Immunocytochemistry. N‐terminal FLAG‐tagged MAB21L1 wild‐type and variants were transfected into HLE‐B3 cells and stained for FLAG (green) and 4ʹ,6‐diamidino‐2‐phenylindole, dihydrochloride (DAPI) (blue; cell nuclei). Wild‐type and variant proteins can be found within the cell nucleus, indicating no disruption in localization. (d) In vivo complementation assays. Proportion of phenotypically normal embryos at 24 hpf in the progeny of heterozygous *mab21l2* ^Q48Sfs*5^ crosses injected with wild‐type or variant Arg51Leu, Arg62Cys, or Gly220Arg *MAB21L1* messenger RNA. UN, uninjected; hpf, hours postfertilization; 1‐MAB21L1‐WT; 2‐MAB21L1‐Arg51Leu; 3‐MAB21L1‐Arg62Cys; 4‐MAB21L1‐Gly220Arg. Statistical significance is indicated by asterisks; *p ≤ .05, **p ≤ .01, and ***p ≤ .001; error bars indicate SEM

The stability and localization of wild‐type and mutant proteins were tested by expressing FLAG‐tagged WT, p.(Arg51Leu), p.(Arg62Cys), and p.(Gly220Arg) constructs in human lens epithelial cells. To assess the stability of the recombinant protein, ELISA and Western blot analysis were used (Figure 2a,b). Both MAB21L1‐p.(Arg62Cys) and p.(Gly220Arg) exhibited reduced protein levels compared with wild‐type, whereas MAB21L1‐p.(Arg51Leu) conversely showed an increase in protein level, suggesting increased stability for this mutant. Quantification using ELISA confirmed these observations and determined that p.(Arg62Cys) and p.(Arg51Leu) levels were significantly different from wild‐type levels (*p* = .012 and *p *= .0074, respectively); however, the p.(Gly220Arg) decrease was not statistically significant (*p *= .068) (Figure 2b).

Next, we investigated the localization of the wild‐type and mutant protein within the cell. Previous studies have found the Mab21l1 protein localizes to the cell nucleus (Mariani et al., 1999). Similarly, our immunofluorescence experiments showed wild‐type MAB21L1 protein within cell nuclei. For all three mutant proteins, no disruption of nuclear localization was seen (Figure 2c). To note, for both wild‐type and mutants, staining was detected in the cytoplasm along with the nucleus. The cytoplasmic staining may be a result of overexpression of our transfected constructs or possibly an indication of nuclear‐cytoplasmic shuttling.

### 3.4 *MAB21L1* wild‐type and mutant mRNA complementation assays in zebrafish *mab21l2* ^Q48Sfs*5^ mutant

To further investigate the effect of the variants, in vivo mRNA complementation assays were performed by injecting mRNA encoding for either MAB21L1 wild‐type or the p.(Arg51Leu), p.(Arg62Cys), or p.(Gly220Arg) variant proteins in equal amounts into one‐ to four‐cell zebrafish embryos generated by *mab21l2* ^Q48Sfs*5^ heterozygous crosses. The *mab21l2* ^Q48Sfs*5^ line (Deml et al., 2015) was used in this experiment because (1) homozygous *mab21l2*‐deficient embryos have an obvious and fully‐penetrant ocular phenotype (microphthalmia with small/absent lens) beginning at 24 hpf (Deml et al., 2015); (2) the underlying mutation causes loss‐of‐function of mab21l2; (3) injections of mRNA encoding for wild‐type MAB21L2 protein were shown to rescue the mutant phenotype (Deml et al., 2015); (4) MAB21L/mab21l proteins are extremely conserved with 94.4% identity between human MAB21L1 and MAB21L2 and 98% identity between human/zebrafish MAB21L1/mab21l1 or MAB21L1/mab21l2 (Figure S3), suggesting a high degree of functional redundancy.

In accordance with Mendel's principles, heterozygous *mab21l2* ^Q48Sfs*5^ parents are expected to produce 75% phenotypically normal embryos comprising a mix of heterozygous (p.Gln48Serfs*5/+) (50%) and wild‐type (+/+) (25%) genotypes and 25% homozygous (p.Gln48Serfs*5/p.Gln48Serfs*5) fish that exhibit the ocular phenotype (Deml et al., 2015). Consistent with this, 77.56% ± 0.27% (number of clutches = 3; total number of embryos per clutch: 131, 75, and 40) of the uninjected progeny of heterozygous crosses had normal eyes. In comparison to uninjected embryos, wild‐type *MAB21L1* mRNA injection (number of clutches = 4; total number of embryos per clutch: 36, 105, 73, and 37) resulted in a significant increase in phenotypically normal embryos (88.38% ± 1.7%; *p *= .0001). This suggests human wild‐type *MAB21L1* is able to functionally substitute for *mab21l2* and rescue the effects of its deficiency (Figure 2d). In contrast, when MAB21L1 p.(Arg51Leu)‐encoding mRNA (number of clutches = 4; total number of embryos per clutch: 49, 92, 52, and 39), p.(Arg62Cys)‐encoding mRNA (number of clutches = 4; total number of embryos per clutch: 102, 53, 129 and 70), or p.(Gly220Arg)‐encoding mRNA (number of clutches = 4; total number of embryos per clutch: 146, 14, 93, and 93) was injected, 78.52% ± 3.8% (*p* = .6922), 82.62% ± 4.4% (*p *= .1100), or 81.40% ± 4.4% (*p* = .2030) of embryos, correspondingly, were phenotypically normal, indicating no statistically significant rescue in comparison to uninjected control had occurred. The lack of rescue was strongest for p.(Arg51Leu). Similarly, mRNA encoding for MAB21L2 Arg51 variant, p.(Arg51Gly), was unable to rescue the *mab21l2* ^Q48Sfs*5^ phenotype (Deml et al., 2015).

## 4 DISCUSSION

In this study, we present four individuals with unique heterozygous coding *MAB21L1* variants, p.(Arg51Leu), p.(Arg62Cys), and p.(Gly220Arg), exhibiting microphthalmia in all, along with variable aniridia, coloboma, microcornea, lens defects (microspherophakia, cataracts) and nystagmus. Previously, homozygous *MAB21L1* variants (mainly truncations) in humans have been associated with a syndromic disorder including ocular features, primarily corneal dystrophy/opacities and nystagmus with variable additional eye anomalies, as well as facial dysmorphism, genital abnormalities, and cerebellar hypoplasia (Bruel et al., 2017; Rad et al., 2019). The data presented in this report suggest that heterozygous missense variants in *MAB21L1* may also be disease‐causing and extend the associated disease spectrum.

All three missense variants were identified in patients with microphthalmia. The c.152G>T p.(Arg51Leu) variant in individuals 1A and 1B was also associated with aniridia and represents the strongest likely causative allele identified in our study, with high functional predictions and complete absence in the general population; in vitro and in vivo studies identified normal localization but higher stability and inability to functionally rescue *mab21l2* deficiency. In addition, mutations affecting the conserved Arg51 residue represent the most common cause of dominant disease in a closely related protein, *MAB21L2* (Deml et al., 2015; Horn et al., 2015; Rainger et al., 2014). The p.(Arg62Cys) and p.(Gly220Arg) variants identified in individuals 2 and 3, respectively, were additionally associated with coloboma; both missense variants demonstrated high functional predictions and inability to effectively rescue *mab21l2* deficiency but as one variant was inherited from an unaffected parent and both variants, though ultra‐rare, were present in the general population, they are currently classified as variants of uncertain significance.

In silico analyses predicted some local structural alterations of variant MAB21L1 proteins. The MAB21L1 structure has two lobes, an N‐terminal and C‐terminal, connected by a long α‐helix spine. The N‐terminal lobe is largely surface‐accessible and contains several positively charged residues, along with a subdomain structurally similar to the NTase domain of cGAS (de Oliveira Mann et al., 2016), which is involved in the recognition of cytosolic nucleic acid and subsequent production of 2ʹ,3ʹ‐cGAMP (Ablasser et al., 2013; Gao et al., 2013). Notably, all three affected residues, Arg51, Arg62, and Gly220, are in or near the N‐terminal lobe upon folding. Arg51 is involved in the formation of salt bridges with residue Glu115, and likely important for protein stabilization (de Oliveira Mann et al., 2016). Arg62 was found to be involved in binding CTP in a denoted “ligand‐binding pocket” outside of the NTase domain (de Oliveira Mann et al., 2016). Though it is unclear whether CTP is a true physiological ligand for MAB21L1, this may highlight the importance of this region for forming interactions and the involvement of Arg62. Finally, Gly220 is located near the N‐terminal lobe upon folding, in a linker region between β8 and β9. Upon mutation to arginine, a gain of an α‐helix is noted, however, it is unclear what the functional consequences of this might be.

Further examination of the identified coding variants by in vitro studies demonstrated normal subcellular localization but variable stability for the corresponding proteins; p.(Arg62Cys) and p.(Gly220Arg) MAB21L1 proteins showed reduced protein levels (statistically significant for p.(Arg62Cys)), whereas p.(Arg51Leu) demonstrated a significantly higher level of protein compared to wild‐type. There are conflicting reports on how the variants at Arg51 affect the stability of MAB21L proteins. The MAB21L1 crystal structure has been solved and revealed that Arg51 participates in salt‐bridge formation and stabilization of loop structures in the protein, suggesting mutations to this residue would affect stabilization of the protein (de Oliveira Mann et al., 2016). Cyclohexamide protein stability assays for p.(Arg51Gly) in MAB21L2 revealed reduced stability in comparison to wild‐type (Deml et al., 2015). Similarly, thermal shift assays for p.(Arg51Cys) in MAB21L1 had reduced melting points compared to wild‐type, suggesting decreased stability (de Oliveira Mann et al., 2016). Conversely, tetracycline protein stability assays for p.(Arg51Cys) and p.(Arg51His) in MAB21L2 indicated increased protein stability (Rainger et al., 2014). The increased stability of p.(Arg51Leu) reported here may further contribute to the pathogenic effects of this variant. For example, as crystallization data suggest that oligomerization may be part of normal MAB21L1 function (de Oliveira Mann et al., 2016), the variant protein may form nonfunctional but more stable complexes with wild‐type MAB21L1, thus exerting a dominant‐negative effect. Another possibility is that increased stability may lead to abnormal persistence of downstream signaling activity; persistent phospho‐ERK signaling has been suggested as a possible pathogenic mechanism for similar MAB21L2 mutants that exhibited increased stability (Rainger et al., 2014).

Finally, mRNA complementation assays were conducted to test the efficiency of *MAB21L1* wild‐type and mutant mRNAs in rescuing a zebrafish *mab21l2* mutant phenotype. Though overexpression of wild‐type *MAB21L1* mRNA compensated for the loss of *mab21l2* and rescued the mutant eye phenotype, similar experiments with mRNA encoding for each of the three mutant proteins failed to produce a statistically significant rescue effect, with p.(Arg51Leu) showing the highest degree of functional deficiency. The observed functional deficiencies of the identified coding variants support their involvement in the corresponding ocular disorders. The presence of the p.(Arg62Cys) and p.(Gly220Arg) variants in the general population (though at ultra‐rare frequencies) and an unaffected parent in one family could be explained by incomplete penetrance. In general, incomplete penetrance has been noted for several genes involved in developmental ocular anomalies. Though pathogenic variants in *OTX2* are an established cause of MAC‐spectrum, review of the literature identified that a number of these variants were inherited from unaffected parents (Schilter et al., 2011). Even more striking, variants in *TEK* associated with primary congenital glaucoma were shown to be inherited from an unaffected parent in all families where both parents were tested (Souma et al., 2016). At the same time, both individuals carrying these variants were found to have additional noncoding *MAB21L1* alleles in *trans* in either the 5ʹ‐UTR (Individual 2; c.‐68T>C) or 3ʹ‐UTR (Individual 3; c.*529A>G). 5ʹ‐UTR variants have the potential to affect translational regulation via impacting the binding of the preinitiation complex, altering recruitment of RNA‐binding proteins (and thus, subsequent events such as RNA capping, splicing, or polyadenylation), or disrupting upstream open reading frames (Steri et al., 2018). 3ʹ‐UTR variants can affect polyadenylation signals, RNA stability and localization, miRNA binding sites, and recruitment of other RNA‐binding proteins involved in translational regulation (Steri et al., 2018). In silico analyses predicted that both the 5ʹ‐ and 3ʹ‐UTR variants identified here affect RBP motifs and the 3ʹ‐UTR allele may have a further effect on miRNA target sites, suggesting both variants could result in aberrant regulation of the *MAB21L1* mRNA. Therefore, it is possible that the noncoding sequence variations affecting the second *MAB21L1* allele in patients carrying p.(Arg62Cys) and p.(Gly220Arg) missense variants contribute to the observed phenotypes in a bi‐allelic manner. Further evidence via identification of other similar families will be needed.

The possible connection of *MAB21L1* with MAC‐spectrum in humans is plausible given the broad expression of this factor in the developing ocular structures in animal models. A *Mab21l1* null mouse displays a severe developmental ocular phenotype consistent with MAC‐spectrum (Yamada et al., 2003). Homozygous embryos fail to form a lens vesicle and exhibit aphakia, malformed retina, and abnormally thick cornea, culminating in severe microphthalmia, disorganized retinal lamination, and highly abnormal anterior structures including absent lens and iris in adults (Yamada et al., 2003). Expression of *Mab21l1* overlaps the developmental pattern of its close homolog, *Mab21l2* (Yamada et al., 2003; Yamada et al., 2004), whose deficiency is also associated with severe ocular defects in mouse (Tsang et al., 2018; Yamada et al., 2004) and zebrafish models (Deml et al., 2015; Gath & Gross, 2019; Hartsock et al., 2014; Wycliffe et al., 2020). Dominant and recessive *MAB21L2* variants have been linked with MAC phenotypes (Aubert‐Mucca et al., 2020; Deml et al., 2015; Horn et al., 2015; Patel et al., 2018; Rainger et al., 2014), with heterozygous alleles affecting the Arg51 residue in four of seven previously published dominant families (c.151C>G p.(Arg51Gly), c.151C>T p.(Arg51Cys) (in two unrelated families), and c.152G>A p.(Arg51His)) and a nearby amino acid at position 49 in another family (c.145G>A p.(Glu49Lys)) (Deml et al., 2015; Horn et al., 2015; Rainger et al., 2014). Additionally, two presumed loss‐of‐function dominant alleles were identified in two unrelated families, c.1A>C p.(Met1?) and c.840C>G p.(Tyr280*) (Aubert‐Mucca et al., 2020; Patel et al., 2018), and one recessive allele was identified in *MAB21L2*, c.740G>A p.(Arg247Gln) (Rainger et al., 2014).

The link between *MAB21L1* and aniridia is also consistent with prior knowledge. The most common cause of aniridia is pathogenic variants in the *PAX6* gene, accounting for up to 90% of cases (Hingorani et al., 2012), with *FOXC1, PITX2*, and a few other genes explaining some of the remaining cases (Hall et al., 2019; Hingorani et al., 2012) but still leaving about 5%–10% of aniridia genetically unexplained. Interestingly, in *Caenorhabditis elegans*, a *mab‐18* mutant (a *PAX6* orthologue) was found to have a very similar phenotype to a *mab‐21* mutant (a *MAB21L* orthologue), both affecting sensory ray formation of the tail (Baird et al., 1991). Furthermore, both *Mab21l1* and *Mab21l2* have previously been suggested as downstream targets of Pax6 in mice. In small‐eye (*sey*) homozygous embryos, in situ hybridization identified a significant reduction in *Mab21l1* expression in both the surface ectoderm and optic vesicles during ocular development, whereas expression of *Mab21l2* in the same tissues was unchanged (Yamada et al., 2003); at the same time, in heterozygous *Pax6* ^*lacZ/+*^ mice (St‐Onge et al., 1997), *Mab21l2* was found to be upregulated in lens tissue implying that *Mab21l2* expression may be normally repressed via Pax6 in the lens (Wolf et al., 2009). Pax6 binding sites were identified in the regulatory regions of *Mab21l2* (Wolf et al., 2009), which further corroborates this interaction and suggests a direct effect. Furthermore, though variants in *PAX6* are typically connected with aniridia, a small number of bilateral and unilateral microphthalmia/coloboma cases have been identified, with or without aniridia (Williamson & FitzPatrick, 2014). The missense variants reported in this manuscript suggest a role for *MAB21L1* in microphthalmia, aniridia, and coloboma in humans, similar to the *PAX6* spectrum. Thus, this study provides additional support for the likely involvement of MAB21L1 and PAX6 in the same pathway. Further genetic screening for *MAB21L1* variants in a wide spectrum of ocular disorders will help to define its role in human eye development and disease.

## WEB RESOURCES

gnomAD Browser: https://gnomad.broadinstitute.org/

Ensembl Variant Effect Predictor: http://uswest.ensembl.org/Tools/VEP

UCSC Genome Browser: https://genome.ucsc.edu/

Kalign: https://www.ebi.ac.uk/Tools/msa/kalign

ViennaRNA Web Services: http://rna.tbi.univie.ac.at/rna.tbi.univie.ac.at/

MicroSNiPer: http://vm24141.virt.gwdg.de/services/microsniper/

PolymiRTS Database 3.0: http://compbio.uthsc.edu/miRSNP/

RBPmap: http://rbpmap.technion.ac.il/index.html

FATHMM‐MKL: http://fathmm.biocompute.org.uk/fathmmMKL.htm

RCSB Protein Data Bank: www.rcsb.org

I‐TASSER: https://zhanglab.ccmb.med.umich.edu/I-TASSER/

ELISA Analysis: elisaanalysis.com

## CONFLICT OF INTERESTS

Adi Reich is an employee of GeneDx, Inc. Remaining authors declare that there are no conflicts of interest.

## Supporting information

Supporting information.

## Data Availability

The authors confirm that the data supporting the findings of this study are available within the article and its Supporting Information material. Variants reported in the manuscript have been submitted to LOVD https://www.lovd.nl/MAB21L1 (IDs: 0036029, 00336030 and 00336031).
