# Supplementary material for: Identification of missense MAB21L1 variants in microphthalmia and aniridia
Source: Hum Mutat. 2021 May 24;42(7):877–90. doi: 10.1002/humu.24218 (PMC8238893; doi:10.1002/humu.24218)
Supplement: Supplementary file 1 — Supporting information. [file HUMU-42-877-s001.pdf]

Supplemental Table 1. Predicted miRNA target effects for c.*529A>G variant.

Variant	miRNA	Database	Conservation Score	Functional Effect	Context Score Change
c.*529A>G	**hsa-miR-1231**	PolymiRTS Database 3.0	10	D	-0.246
		MicroSNiPer	N/A	D	N/A
	**hsa-miR-1299**	PolymiRTS Database 3.0	11	D	-0.093
		MicroSNiPer	N/A	D	N/A
	**hsa-miR-6128**	PolymiRTS Database 3.0	11	D	-0.077
	**hsa-miR-654-3p**	PolymiRTS Database 3.0	12	D	-0.137`
		MicroSNiPer	N/A	D	N/A
	**hsa-miR-875-3p**	PolymiRTS Database 3.0	11	D	-0.071
		MicroSNiPer	N/A	C	N/A
	**hsa-miR-3681-5p**	MicroSNiPer	N/A	U	N/A
	**hsa-miR-1254**	PolymiRTS Database 3.0	10	C	-0.392
		MicroSNiPer	N/A	C	N/A
	**hsa-miR-3116**	PolymiRTS Database 3.0	10	C	-0.413
		MicroSNiPer	N/A	C	N/A
	**hsa-miR-661**	PolymiRTS Database 3.0	10	C	-0.15
		MicroSNiPer	N/A	C	N/A
	**hsa-miR-4633-5p**	MicroSNiPer	N/A	C	N/A
	**hsa-miR-4254**	MicroSNiPer	N/A	C	N/A

D: variant disrupts the predicted miRNA site; C: variant creates a predicted miRNA site; U: unaffected.

Supplemental Table 2. Predicted RNA binding protein (RBP) effects with non-coding variation.

Untranslated Region	Variant	RNA-Binding Protein	Motif	Functional Effect	Z-score	P-value
5' UTR	WT	SRSF3	cuckucy	D	1.670	4.75e-02
	c.-68T>C				N/A	N/A
	WT	RBM6	hauccar	C	N/A	N/A
	c.-68T>C				2.775	2.76e-03
3'UTR	WT	HNRNPL	amayama	D	2.387	8.49e-03
	c.*529A>G				N/A	N/A
	WT	HNRPLL	rcahaca	U	2.519	5.88e-03
	c.*529A>G				2.025	2.14e-02
	WT	IGF2BP2	vmahwca	D	1.986	2.35e-02
	c.*529A>G				N/A	N/A
	WT	RBM41	wuacwuk	D	2.420	7.76e-03
	c.*529A>G				N/A	N/A
	WT	SRSF3	wcwwc	D	2.402	8.15e-03
	c.*529A>G				N/A	N/A
	WT	SRSF5	yywcwsg	C	N/A	N/A
	c.*529A>G				2.204	1.38e-02

D: variant disrupts the predicted RBP motif site; C: variant creates a predicted RBP motif; U: unaffected.
Z-score and P-value reflect the reliability of the predicted binding site

Supp. Figure 1. *In silico* protein modeling.

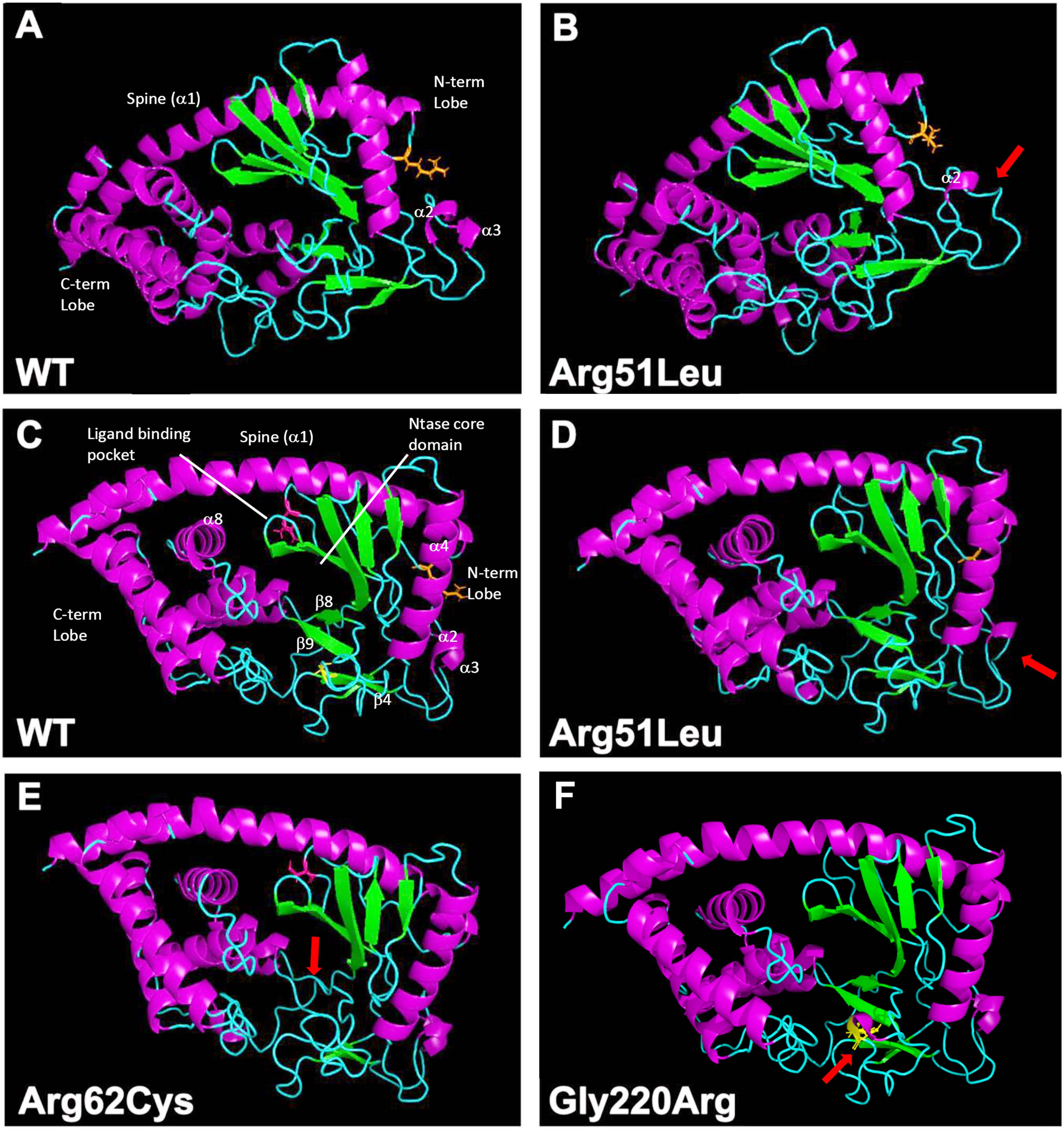

Supp. Figure 1. ***In silico*** **protein modeling.** Protein structures for the solved MAB21L1 wild-type (5EOG) (de Oliveira Mann et al., 2016) (**A,C**) and the I-TASSER predicted mutants (Arg51Leu **B,D**; Arg62Cys **E**; Gly220Arg **F**). Amino acids (aa) of interest are highlighted as follows: aa51 orange, aa62pink, aa220 yellow. Alpha helices are purple, beta sheets green, linker regions blue. Red arrows (**B**,**D**,**E**,**F**) indicate observed structural changes upon mutation.

Supp. Figure 2. Multi-species sequence comparisons of *MAB21L1* non-coding regions.

A **5' UTR sequence;** 13:36050329 – 36050370 (forward sequence)

```
Human                        tttctccaccgaagttggctccagctctagcagccgcattgg
Individual 2                                             c
Chimp                        tttctccaccgaagttggctccagctctagcagccgcattgg
Gorilla                      tttctccaccgaagttggctccagctctagcagccgcattgg
Orangutan                    tttctccaccgaagttggctccagctctagcagccgcattgg
Gibbon                       tttctccaccgaagttggctccagctctagcagccgcattgg
Baboon                       tttctccaccgaagttggctccagctctagcagccgcattgg
Green monkey                 tttctccaccgaagttggctccagctctagcagccgcattgg
Marmoset                     tttctccaccgaagttggctccagctctagcagccgcattgg
Squirrel monkey              tttctccaccgaagttggctccagctctagcagccgcattgg
Chinese tree shrew           tttctccaccgaagttggctccagctctagcagccgcattgg
Squirrel                     tttctccaccgaagttggctccagctctagcagccgcattgg
Lesser Egyptian jerboa       ggttctctccgaagttggctcccgcgccagcagccgcattgg
Prairie vole                 tttcaccaccgaagttggctccagcgctagcagccgcattgg
Chinese hamster              tttcacctccgaagttggctccagcgctagcagccgcattgg
Golden hamster               tttcacctccgaagttggctccagcgctagcagccgcattgg
Mouse                        tttctccaccgaagttggctccagcgctagcagccgcattgg
Rat                          tttctccaccgaagttggctccagcgctagcagccgcattgg
Naked mole-rat               tttctccaccgaagttggctccagctctagcagccgcattgg
Guinea pig                   tttctccaccgaagttggctccagctctagcagccgcattgg
Chinchilla                   tttctccaccgaagttggctccagctctagcagccgcattgg
Brush-tailed rat             attctccaccgaagttggctccagctctagcagccgcattgg
Rabbit                       tttctccaccgaagttggctccagctctagcagccgcattgg
Pika                         tttctccaccgaagttggctccagctctagcagccgcattgg
Pig                          tttctccaccgaagttggctccagctctagcagccgcattgg
Alpaca                       tttctccaccgaagttggctccagctctagcagccgcattgg
Bactrian camel               tttctccaccgaagttggctccagctctagcagccgcattgg
Killer whale                 tttctccaccgaagttggctccagctctagcagccgcattgg
Tibetan antelope             tttctccaccgaagttggctccagctctagcagccgcgttgg
Cow                          tttctccaccgaagttggctccagctctagcagccgcgttgg
Sheep                        tttctccaccgaagttggctccagctctagcagccgcgttgg
Domestic goat                tttctccaccgaagttggctccagctctagcagccgcgttgg
Horse                        tttctccaccgaagttggctccagctctagcagccgcattgg
White rhinoceros             tttctccaccgaagttggctccagctctagcagccgcattgg
Cat                          tttctccaccgaagttggctcccgctctagcagccgcattgg
Ferret                       tttctccaccgaagttggctccagcgctagcagccgcattgg
Panda                        tttctccaccgaagttggctccagcgctagcagccgcattgg
Pacific walrus               tttctccaccgaagttggctccagctctagcagccgcattgg
Weddell seal                 tttctccaccgaagttggctccagctctagcagccgcattgg
Megabat                      tttctgcac-gaagttggctccagccccagcagccgcattgg
David's myotis (bat)         tttctgcgc-gaagttggctccagctctagcagccgcattgg
Microbat                     tttctgcac-gaagttggctccagctctagcagccgcattgg
Big brown bat                ttcctgcag-gaagttggctccggc-cgggccgccgcgttgg
Hedgehog                     tttctccaccgaagttggctccagcgctagcagccgcattgg
Shrew                        tttctccaccgaagttggctccagcgctagcagccgcattgg
Star-nosed mole              tttctccaccgaagttggcgccagctctagcagccgcattgg
Cape elephant shrew          tttctcccccgaagttggctcccgcgccagcagccgcattgg
Manatee                      tttc--ccccgaagttggctccagcgccagcagccgcattgg
Cape golden mole             ttcctccccaaagttggctcccgcgctggcagccgcgttgg
Tenrec                       gtcctcctccgaagttggctcccgcgctggcagccgcgttgg
Aardvark                     attctgcccgaagttggcgccagcgctcgcagccgcattgg
Armadillo                    ccccggcacccgcgttggctcccgcgcccgcagccgcattgg
Opossum                      tttctcctctgaagttggctccagctttagcagccgcattgg
Tasmanian devil              tttctcctctgaagttggctccagctttagcagccgcattgg
Wallaby                      tttctcctctgaagttggctccagctttagcagccgcattgg
Platypus                     tttctcctctgaagttggctcccgcctcagcagccgcattgg
Saker falcon                 tttctcctctgaagttggctccagccttagcagccgcattgg
Peregrine falcon             tttctcctctgaagttggctccagccttagcagccgcattgg
Collared flycatcher          tttctcctctgaagttggctccagccttagcagccgcattgg
White-throated sparrow       tttctcctctgaagttggctccagccatagcagccgcattgg
Medium ground finch          tttctcctctgaagttggctccagccatagcagccgcattgg
Zebra finch                  tttctcctctgaagttggctccagccatagcagccgcattgg
Tibetan ground jay           tttctcctctgaagttggctccagccttagcagccgcattgg
Budgerigar                   tttctcctctgaagttggctccagccttagcagccgcattgg
Parrot                       tttctcctctgaagttggctccagccttagcagccgcattgg
Scarlet macaw                tttctcctctgaagttggctccagccttagcagccgcattgg
Rock pigeon                  tttctcctctgaagttggctccagccttagctgccgcattgg
Mallard duck                 tttctcctctgaagttggctccagccttagcagccgcattgg
Chicken                      attctcccc-gaagttggctcccgccttagcagccgcattgg
American alligator           tttctcccctgaagttggctccagccctagcagccgcattgg
Green seaturtle              tttcccctctgaagttggctccagccttagcagccgcattgg
Painted turtle               tttcccctctgaagttggctccagccttagcagccgcattgg
Chinese softshell turtle     tttctcctctgaagttggctccagccttagcagccgcattgg
Lizard                       gattcccgctgccgccgccccgcccctgccagccgcgttgg
Coelacanth                   tatttttcctgaagttggctccagtcaaattagccgcattgg
Tetraodon                    tttcttctctaaaattggctcccgtacaaacagccgcgttgg
Fugu                         tttcttctctaaaattggctcccgtacaaacagccgcgttgg
Yellowbelly pufferfish       tttcttctctaaaattggctcccgtacaaacagccgcgttgg
Nile tilapia                 tttcttctctaaaattggctcccgtacaaacagccgcgttgg
Zebra mbuna                  tttcttctctaaaattggctcccgtacaaacagccgcgttgg
Medaka                       cttcttctctaaaattggctcccgtacaaacagccgcgttgg
Southern platyfish           cttcttctctaaaattggctcccgtacaaacagccgcgttgg
Stickleback                  tttcttctctaaaattggctcccatacaaacagccgcgttgg
Atlantic cod                 cttcttctcttaaattggctcccgtacaaaccgccgcgttgg
Zebrafish                    attcttctctaaaattggctcctgtacaaacagccgcgttgg
Mexican tetra (cavefish)     tttcttctctaaagttggctcccgcgcccacggccgcgttgg
Lamprey                      ttctttcgctg----cgctgcccgcccacccgttcgcgttgg
```

B **3' UTR sequence;** 13:36048652 – 36048681 (forward sequence)

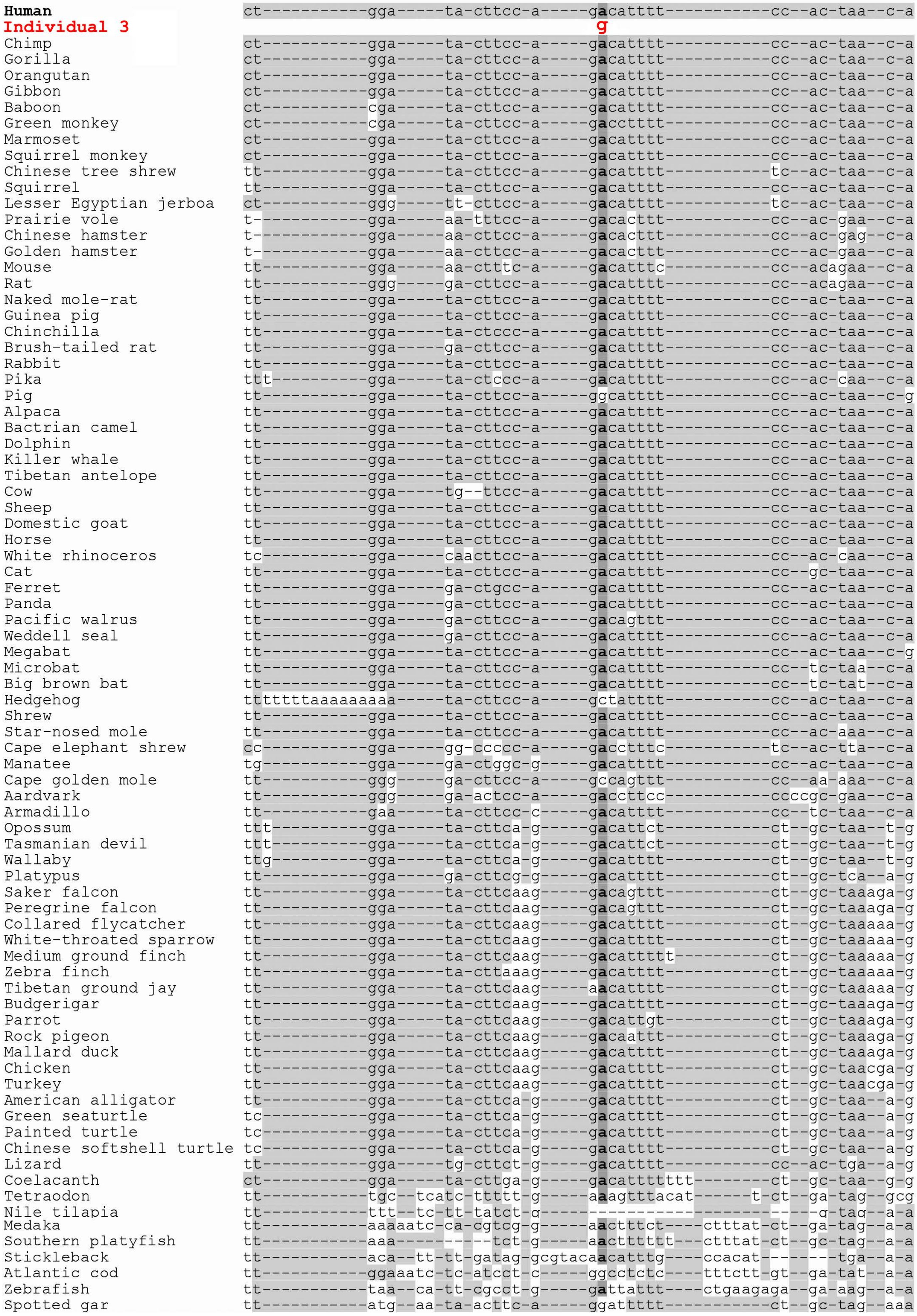

Supp. Figure 2. Multi-species sequence comparisons of *MAB21L1* non-coding regions.

Nucleotide alignments for 5’UTR (**A**) and 3’UTR (**B**) are shown using data generated by UCSC Multiz Alignments of 100 Vertebrates. Identical nucleotides are shaded in grey; positions of variant nucleotides are indicated in bold; non-coding variants in Individuals 2 (**A**) and 3 (**B**) are shown in red.

Supp. Figure 3. Alignment of full-length MAB21L1 and related proteins.

```
MAB21L1             MIAAQAKLVYHLNKYYNEKCQARKAAIAKTIREVCKVVSDVLKEVEVQEPRFISSLNE-M  59
MAB21L2             MIAAQAKLVYQLNKYYTERCQARKAAIAKTIREVCKVVSDVLKEVEVQEPRFISSLSE-I
mMab21l1            MIAAQAKLVYHLNKYYNEKCQARKAAIAKTIREVCKVVSDVLKEVEVQEPRFISSLNE-M
cMab21l1            MIAAQAKLVYHLNKYYNEKCQARKAAIAKTIREVCKVVSDVLKEVEVQEPRFISSLNE-M
mab21l1             MIAAQAKLVYHLNKYYNEKCQSRKAAISKTIREVCKVVSDVLKEVEVQEPRFISSLNE-M
mab-21              MLGHNQNVVYQVNNYFNEKVQHRKVRVTKTVQRIAKVVQEILKEVEAQEPRFINTLSETT
Individuals 1a, 1b                                                   L

MAB21L1             DNRYEGLEVI-SPTEFEVVLYLNQMGVFNFVDDGSLPGCAVLKLSDGRKRSMSLWVEFIT  118
MAB21L2             DARYEGLEVI-SPTEFEVVLYLNQMGVFNFVDDGSLPGCAVLKLSDGRKRSMSLWVEFIT
mMab21l1            DNRYEGLEVI-SPTEFEVVLYLNQMGVFNFVDDGSLPGCAVLKLSDGRKRSMSLWVEFIT
cMab21l1            DNRYEGLEVI-SPTEFEVVLYLNQMGVFNFVDDGSLPGCAVLKLSDGRKRSMSLWVEFIT
mab21l1             DNRFEGLEVI-SPTEFEVVLYLNQMGVFNFVDDGSLPGCAVLKLSDGRKRSMSLWVEFIT
mab-21              TGRFDGI-VVHSPSEYEAVLYLNQMGVFNFVDDGTIQGCAVLKLSDGRKRSMSLWVEFIT
Individual 2          C

MAB21L1             ASGYLSARKIRSRFQTLVAQAV------DKCSYRDVVKMVADTSEVKLRIRDRYVVQITP  172
MAB21L2             ASGYLSARKIRSRFQTLVAQAV------DKCSYRDVVKMIADTSEVKLRIRERYVVQITP
mMab21l1            ASGYLSARKIRSRFQTLVAQAV------DKCSYRDVVKMVADTSEVKLRIRDRYVVQITP
cMab21l1            ASGYLSARKIRSRFQTLVAQAV------DKCSYRDVVKMVADTSEVKLRIRDRYVVQITP
mab21l1             ASGYLSARKIRSRFQTLVAQAV------DKCSYRDVVKMVADTSEVKLRIRDRYVVQITP
mab-21              ASGYLSARKIRHRFQNIVAQVLQTPQFSDYC------KLLQDNTDVRVRVDDKYTVQITC

MAB21L1             AFKCTGIWPRSAAHWPLPHIPWPGPNR-VA-EVKAEGFNLLSKECHSLA-----GKQSSA  225
MAB21L2             AFKCTGIWPRSAAQWPMPHIPWPGPNR-VA-EVKAEGFNLLSKECYSLT-----GKQSSA
mMab21l1            AFKCTGIWPRSAAHWPLPHIPWPGPNR-VA-EVKAEGFNLLSKECHSLA-----GKQSSA
cMab21l1            AFKCTGIWPRSAAHWPLPHIPWPGPNR-VA-EVKAEGFNLLSKECHSLA-----GKQSSA
mab21l1             AFKCTGIWPRSAAHWPLPHIPWPGPNR-VA-EVKAEGFNLLSKECYSLN-----GKQSSA
mab-21              AFRCNGIWPRSASHWPIAGLPWP--NAALANQTKAEGFDLTSRET-AITQQNNPNKQASS
Individual 3                                                             R

MAB21L1             -ESDAWVLQFAEAENRLQMGGCRKKCLSILKTLRDRHLELPGQPLNNYHMKTLVSYECEK  284
MAB21L2             -ESDAWVLQFGEAENRLLMGGCRNKCLSVLKTLRDRHLELPGQPLNNYHMKTLLLYECEK
mMab21l1            -ESDAWVLQFAEAENRLQMGGCRKKCLSILKTLRDRHLELPGQPLNNYHMKTLVSYECEK
cMab21l1            -ESDAWVLQFAEAENRLQMGGCRKKCLSILKTLRDRHLELPGQPLNNYHMKTLVSYECEK
mab21l1             -ESDAWVLQFAEAENRLLLGGCRKKCLSLLKTLRDRHLELPGQPLNNYHMKTLVSYECEK
mab-21              MEADAWAMKMHGAENMLLTGG-RRKTLSILKCLRDAHMDFPGTPVTNYILKTLVLYECEK

MAB21L1             HPRESDWDESCLGDRLNGILLQLISCLQCRRCPHYFLPNLDLFQGKP-HSALENAAKQTW  343
MAB21L2             HPRETDWDESCLGDRLNGILLQLISCLQCRRCPHYFLPNLDLFQGKP-HSALESAAKQTW
mMab21l1            HPRESDWDESCLGDRLNGILLQLISCLQCRRCPHYFLPNLDLFQGKP-HSALENAAKQTW
cMab21l1            HPRESDWDESCLGDRLNGILLQLISCLQCRRCPHYFLPNLDLFQGKP-HSALENAAKQTW
mab21l1             HPRESDWDENCLGDRLNGILLQLISCLQCRRCPHYFLPNLDLFQGKP-HSALENAAKQTW
mab-21              HCSEYEWEDPNIGDRLVGILLQLVSCLQCRRCAHYFLPSLDLLRSKPVHS-IEHSAQLAW

MAB21L1             RLAREILTNPKSLEKL  359
MAB21L2             RLAREILTNPKSLDKL
mMab21l1            RLAREILTNPKSLEKL
cMab21l1            RLAREILTNPKSLEKL
mab21l1             RLAREILTNPKSLEKL
mab-21              HLVRKLMIDPNALQSL
```

Supp. Figure 3. Alignment of full-length MAB21L1 and related proteins. Identical amino acids are shaded in grey; positions of variant amino acids are indicated in bold and variants identified in Individuals 1a, 1b, 2 and 3 are shown in red. Note high conservation between different vertebrate MAB21L1/Mab21l1/mab21l1 proteins as well as with its close human homolog MAB21L2 and *C. elegans* mab-21. Human MAB21L1 (NP_005575.1), human MAB21L2 (NP_006430.1), mouse Mab21l1 (NP_034880.1), chicken Mab21l1 (NP_989864.1), zebrafish mab21l1 (NP_694506.2) and *C. elegans* mab-21 (NP_497940.2).
